# Thyroid Eye Disease and Glaucoma: A Cross-Sectional Study Comparing Clinical Characteristics and Disease Severity

**DOI:** 10.3390/medicina60091430

**Published:** 2024-09-01

**Authors:** Laura Andreea Ghenciu, Alina Maria Șișu, Emil Robert Stoicescu, Alexandra-Ioana Dănilă, Roxana Iacob, Mihai-Alexandru Săndesc, Ovidiu Alin Hațegan

**Affiliations:** 1 Department of Functional Sciences, ‘Victor Babes’ University of Medicine and Pharmacy Timisoara, Eftimie Murgu Square No. 2, 300041 Timisoara, Romania; 2 Center for Translational Research and Systems Medicine, ‘Victor Babes’ University of Medicine and Pharmacy Timisoara, Eftimie Murgu Square No. 2, 300041 Timisoara, Romania; 3 Department of Anatomy and Embriology, ‘Victor Babes’ University of Medicine and Pharmacy Timisoara, 300041 Timisoara, Romania; alexandra.danila@umft.ro (A.-I.D.); roxana.iacob@umft.ro (R.I.); 4 Field of Applied Engineering Sciences, Specialization Statistical Methods and Techniques in Health and Clinical Research, Faculty of Mechanics, ‘Politehnica’ University Timisoara, Mihai Viteazul Boulevard No. 1, 300222 Timisoara, Romania; 5 Department of Radiology and Medical Imaging, ‘Victor Babes’ University of Medicine and Pharmacy Timisoara, Eftimie Murgu Square No. 2, 300041 Timisoara, Romania; 6 Research Center for Pharmaco-Toxicological Evaluations, ‘Victor Babes’ University of Medicine and Pharmacy Timisoara, Eftimie Murgu Square No. 2, 300041 Timisoara, Romania; 7 Doctoral School, “Victor Babes” University of Medicine and Pharmacy Timisoara, Eftimie Murgu Square 2, 300041 Timisoara, Romania; 8 Department of Orthopedics and Traumatology, “Victor Babes” University of Medicine and Pharmacy, Timisoara, Eftimie Murgu Square No. 2, 300041 Timisoara, Romania; sandesc.mihai@umft.ro; 9 Discipline of Anatomy and Embriology, Medicine Faculty, Vasile Goldis” Western University of Arad, Revolution Boulevard 94, 310025 Arad, Romania; hategan.ovidiu@uvvg.ro

**Keywords:** thyroid eye disease, Graves’ ophthalmopathy, open-angle glaucoma

## Abstract

*Background and Objectives*: This study investigates the relationship between thyroid eye disease (TED) and open-angle glaucoma (OAG), focusing on disease severity and clinical features. *Materials and Methods*: Conducted at the Timis County Emergency Clinical Hospital, the research included 106 patients, with 53 having both conditions and 53 having only OAG. Key metrics analyzed included intraocular pressure (IOP) using a Goldmann applanation tonometer, the retinal nerve fiber layer (RNFL) thickness, and optic nerve head (ONH) characteristics evaluated using optical coherence tomography (OCT). *Results*: Results indicated that patients with both TED and OAG experienced a 6.09% reduction in RNFL thickness and showed more rapid disease progression, with 48.35% having active TED. The mean IOP in TED patients was 27.5 ± 4.9 mmHg, which was similar to those with only OAG. Demographic factors, including age and gender, influenced the clinical course and disease severity. *Conclusions*: These findings underscore the importance of specialized monitoring and treatment strategies for patients with coexisting TED and OAG to prevent vision loss.

## 1. Introduction

Basedow–Graves’ disease is an autoimmune disorder characterized by hyperthyroidism due to thyroid-stimulating immunoglobulins (TSIs) that stimulate the gland to produce excessive thyroid hormones. This condition can lead to a state of thyrotoxicosis, with clinical manifestations including diffuse goiter, weight loss, heat intolerance, tachycardia, and tremors. Laboratory findings typically show elevated levels of free thyroxine (fT4) and triiodothyronine (fT3) with suppressed thyroid-stimulating hormone (TSH) [1]. Epidemiologically, it is more prevalent in women, with a higher incidence in middle-aged individuals. This condition poses significant public health challenges due to its impact on metabolic rate and potential complications if untreated [1,2]. The pathogenesis of Basedow–Graves’ disease involves a complex relation between genetic and environmental factors, resulting in the loss of immune tolerance and the production of autoantibodies against TSH receptors.

Thyroid eye disease (TED), also known as Graves’ ophthalmopathy, is often associated with Basedow–Graves’ disease. It is characterized by inflammation and remodeling of the extraocular muscles and orbital connective tissue, leading to symptoms such as proptosis, periorbital edema, diplopia, and, in severe cases, compressive optic neuropathy. The pathophysiology involves the activation of orbital fibroblasts by autoantibodies targeting the TSH receptor, resulting in the secretion of glycosaminoglycans and cytokines that contribute to tissue expansion and fibrosis [3]. Management of TED includes measures to control thyroid dysfunction, immunosuppressive therapies (e.g., corticosteroids, rituximab), and, in severe cases, surgical interventions, such as orbital decompression, strabismus surgery, and eyelid surgery, to alleviate symptoms and prevent vision loss [4]. Early diagnosis and a multidisciplinary approach are essential for optimal outcomes.

Glaucoma is one of the key contributors to permanent visual disability worldwide. Despite the fact that glaucoma is caused by a multitude of variables, the primary goal of management is to consistently lower intraocular pressure (IOP) through medication or surgery [5]. One major risk factor for glaucoma is elevated IOP; studies have been done on the incidence of open-angle glaucoma (OAG) or intraocular hypertension in individuals with TED, but the findings are debatable [6]. Although there is evidence linking TED to ocular hypertension [7], glaucoma prevalence does not appear to be substantially higher in TED patients than in individuals without TED [8,9]. Previous research indicates that OAG affects 0.8–13.5% of TED patients [10,11]. Few papers currently exist describing increased IOP in TED utilizing the EUGOGO system. Clinical aspects of high IOP have primarily been studied using the NOSPECS categorization of TED [12]. Treating gaze-dependent IOP rises in TED is often deemed unnecessary [7]. Meanwhile, an elevation in IOP may result from using corticosteroids to treat the intraorbital inflammatory component of the disease [7]. Even though some research has indicated that orbital decompression can lower IOP [13], this step is essential for lowering episcleral venous pressure in patients needing filtering surgery. Orbital decompression also addresses critical issues such as dysthyroid optic neuropathy, exposure keratopathy, and severe proptosis in TED. This approach aims to reduce the risk of suprachoroidal hemorrhage and choroidal effusion prior to or following glaucoma surgical procedures. Increased retrobulbar pressure, enlarged and fibrous extraocular muscles, glycosaminoglycan accumulation in the trabecular meshwork, genetic susceptibility to thyroid disorders and glaucoma, and elevated episcleral venous pressure as a result of orbital congestion are all potential causes of elevated IOP [14,15,16]. Additionally, glaucoma may have been present before the onset of thyroid disease, or the thyroid condition itself could be a risk factor for developing glaucoma. This suggests a complex relationship where TED may contribute to or exacerbate existing ocular conditions. Metabolic and endocrinologic diseases are closely linked to various eye diseases, affecting visual health and potentially leading to conditions like retinopathy and glaucoma [7,17,18].

Our research aimed to investigate the differences between OAG patients with and without concurrent TED, rather than exploring the underlying mechanisms or causes of glaucoma. Specifically, we focused on comparing the severity and clinical features of OAG in these two patient groups.

## 2. Materials and Methods

We carried out a retrospective hospital-based study at the Timis County Emergency Clinical Hospital from 1 January 2015 to 31 December 2020. During this study period, all patients aged 18 years or over with glaucoma were eligible for inclusion in this study if they were diagnosed according to the criteria described in Table 1. This cross-sectional study was conducted following the Declaration of Helsinki and approved by the Ethics Committee of the Timis County Emergency Clinical Hospital (protocol code 412/31.10.2023). The medical records of patients with Basedow–Graves’ disease who came for an ophthalmology consult were reviewed. Basedow–Graves’ disease was diagnosed in the Department of Endocrinology based on clinical findings, thyroid hormones (fT3 and fT4), thyroid-stimulating hormone (TSH), and thyroid antibodies (TRAb and TSAb). In diagnosing TED, we focused on key clinical signs such as eyelid retraction, which was the most prevalent (84.9%). Other important signs included proptosis, conjunctival injection, lid lag on downward gaze and/or lid edema, and restricted eye movement due to enlarged extraocular muscles. TED was assessed and classified in the Department of Ophthalmology, analyzing the duration of orbitopathy and thyroid disease, therapy of thyroid disease, and the index of disease activity based on a clinical activity score (CAS); we also assessed the severity of TED by European Group on Graves’ Orbitopathy (EUGOGO) classification [19].

We collected all available data, including applanation tonometry, autorefractometry, exophthalmometry, and funduscopy data. OAG was defined by the presence of the two following parameters: an IOP ≥ 21 mmHg in the presence of an open angle on gonioscopy in either one or both eyes and signs indicative of glaucoma in the optic nerve head (ONH). Exclusion criteria included age < 18, refractive error > ±6 diopters and/or astigmatism > ±2 diopters, previous ocular surgery, closed angle on gonioscopy, and other autoimmune disorders. The collected data comprised age, gender, and systemic and local signs and symptoms (CAS and EUGOGO). Investigations were also conducted on the potential presence of risk factors, including history of smoking, ocular or systemic disorders, and family history for the development of open-angle glaucoma in patients with TED. The anterior chamber was visualized under a slit-lamp, IOP was measured with Goldmann applanation tonometry (for patients with corneal impairment, IOP was measured appropriately with a Pascal dynamic contour tonometer), proptosis was assessed with a Hertel exophthalmometer, the irido-corneal angle was assessed using a Goldmann three-mirror lens, and Cirrus HD-OCT 500 (Carl Zeiss Meditec, Inc., Dublin, CA, USA) measurements were conducted. We analyzed the structural damage of 12 sections of the clock hour retinal nerve fiber layer on the OCT. A cup-to-disc ratio above 0.6, a vertical cup asymmetry above 0.2, the presence of neuroretinal rim loss or notching accompanied or unaccompanied by disc hemorrhages, and RNFL abnormalities were considered to be indicators of glaucomatous alterations in the optic nerve for both the TED + OAG and OAG group.

The CAS was used to measure disease activity in group A. It is a 7-point binary grading system that takes into account soft tissue indicators and symptoms that point to anterior orbital inflammation. A CAS ≥ 3 during presentation indicates active TED. This includes spontaneous retrobulbar pain, pain with ocular movement, eyelid hyperemia, conjunctival hyperemia, swelling of the caruncle/plica, swelling of the eyelids, and chemosis [20]. We used the EUGOGO classification to evaluate the severity and activity of the disease. Reduced monocular movement and declining best-corrected visual acuity (BCVA) were used to grade function, while four indicators of inflammation—discomfort, redness, swelling, and decreased function—were used to determine activity. We categorized the patients as having mild, moderate–severe, and sight-threatening disease depending on the EUGOGO system. The criteria for a mild illness included mild lid retraction (less than 2 mm), minor soft tissue involvement, exophthalmos (less than 3 mm above normal), minimal or sporadic diplopia, and lubricant-responsive exposure keratopathy. The criteria for moderate–severe TED included lid retraction of at least 2 mm, moderate to severe soft tissue involvement or exophthalmos of at least 3 mm above the normal value for both gender and race, and fluctuating or continuous diplopia. Thyroid-related optic neuropathy, important keratopathy, or corneal breakdown (descemetocele or open perforation) were considered indicators of sight-threatening TED.

### Statystical Analysis

We conducted the analyses using SPSS 29.0 (Statistical Package for Social Sciences; SPSS Inc. IBM, North Castle, NY, USA). The analyzed data are expressed as means ± standard deviation. T-tests were used to determine the relevance of differences between the two groups. At *p* < 0.05, differences were deemed statistically significant. We also used two-way ANOVAs to investigate the differences in IOP between the groups and across age categories. To compare the distribution of categorical variables between the two groups, we utilized a chi-square test.

## 3. Results

### 3.1. Demographic Characteristics

The analyses comprised 106 subjects in total, who were included in the normative database. Out of the total patients observed in the study, 53 patients (91 eyes) were diagnosed with TED and OAG (group A), and the other 53 patients (88 eyes) were diagnosed with OAG with no other thyroid-related diseases (group B). Table 2 summarizes the clinical and demographic patient characteristics, including age, gender, smoking behaviors, systemic illnesses, and TED course. The vast majority of patients in group A were females, while group B exhibited a very small difference between the number of males and females. The average age of the patients in group A was 39.70 ± 9.06 years, with no significant difference in mean age between male (38.5 ± 9.2 years) and female (39.70 ± 8.9 years) patients. In group B, the average age of patients was 55.80 ± 7.09 years. Between the two groups, as expected, there was a significant difference in age between group A and group B (T-statistic: −12.842, *p* < 0.001).

The treatment distribution among patients shows that the majority (75.40%, n = 40) were on antithyroid medications, while 13.20% (n = 7) underwent thyroidectomy, and 11.30% (n = 6) received radioiodine therapy. A chi-square test was applied to evaluate the distribution of these treatments, but the results did not show any significant differences between the treatment modalities (*p* > 0.05). Additionally, at the time of ocular examination, only 15.10% (n = 8) of patients had normal thyroid function, indicating that a significant proportion (84.90%, n = 45) remained hyperthyroid. This imbalance in thyroid function status was statistically significant (*p* < 0.01).

Smoking was equally distributed throughout the two groups, as seen by the variations in risk variables between people in group A and B (56.60% vs. 52.83%). Diabetes mellitus was almost equally present in both groups (7.54% vs. 9.43%), but arterial hypertension was found to have a higher prevalence in group A (47.16% vs. 30.18%). Other comorbidities such as hyperlipidemia, depression, asthma, or chronic obstructive pulmonary disease were absent in the majority of patients in both groups. About one-third of patients from group A (30.18%) had a positive family history for thyroid-related disease, and less than 10% of patients had a positive family history for OAG plus thyroid gland disease. To compare the distribution of categorical variables between group A and group B, we utilized a chi-square test. This statistical test was chosen to determine if there were significant differences between the groups for smoking behavior, arterial hypertension, and diabetes mellitus. For smoking behavior, arterial hypertension, and diabetes mellitus, the chi-square tests yielded values of 0.152, 3.2218, and 0.1216, respectively, with *p* > 0.05, indicating that none of these results were statistically significant.

### 3.2. Ocular Presentation

The IOP ranged from 21 to 47 mmHg, with a mean of 27.5 ± 4.90 mmHg in group A and 28.1 ± 4.90 mmHg in group B. We analyzed the two groups and concluded that the results of IOP across the groups did not differ statistically (T-statistic: −0.610, *p* = 0.543). Data on IOP and age were collected from patients in two groups, A and B. The patient age was categorized into five age groups: 20–29, 30–39, 40–49, 50–59, and over 60. The pressure values were then statistically analyzed using a two-way ANOVA to investigate the differences in IOP between groups and across age categories. The results indicated that there were no statistically significant differences in IOPs between age groups (*p* > 0.05), between groups A and B (*p* > 0.05), or in the interaction between age group and patient group (*p* > 0.05).

In group A, a T-test comparing the IOP values between smokers (mean = 28.63 ± 6.20) and non-smokers (mean = 28.19 ± 6.31) resulted in a *p*-value greater than 0.05, indicating no statistically significant difference. Conversely, in group B, the t-test comparing smokers (mean = 33.09 ± 4.77) and non-smokers (mean = 25.08 ± 2.60) yielded a *p*-value less than 0.05, revealing a statistically significant difference. This suggests that smoking significantly impacts IOP in group B but not in group A. 

In the two groups under OCT examination, measurements included the average cup-to-disc ratio, neuroretinal rim area thickness, and optic disc surface area. Group A had lower median values of average RNFL thickness, as well as greater average cup-to-disc ratios than group B, but there was no significant difference between them (*p* > 0.05). In comparing RNFL thickness between group A (mean = 70.06 ± 6.95 microns) and group B (mean = 74.60 ± 5.25 microns), a T-test indicated a statistically significant reduction of 6.09% in group A. Similar reduction trends are observed across different RNFL quadrants. Additionally, group A exhibited a thinner neuroretinal rim area compared to group B, though the disc surface area did not differ significantly between the two groups. The ONH variables changed more significantly in the TED group than in the OAG group, highlighting a greater impact in the TED group.

**Figure 1:**
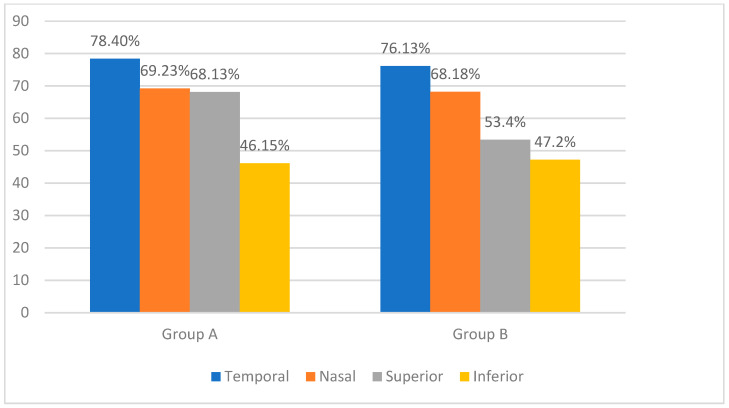
Percentage of patients with retinal nerve fiber layer thinning in different quadrants: comparison between group A and group B.

We combined the clock hours into four quadrants to provide a clear and clinically relevant analysis of nerve fiber layer thinning: superior quadrant (clock hours 10 to 2), inferior quadrant (clock hours 4 to 8), nasal quadrant (clock hours 2 to 4), and temporal quadrant (clock hours 8 to 10). In group A, the temporal sector was the most affected (78.40%), followed by the nasal and superior sectors at almost identical incidences (69.23% and 68.13%), and the inferior sector was the least affected (46.15%). In group B, the temporal (76.13%) and nasal (68.18%) sectors showed important thinning, followed by the superior (53.40%) and inferior (47.20%) sectors. Figure 1 displays the distribution of nerve fiber layer thinning in the database subjects as determined by Cirrus SD HD-OCT.

**Figure 2:**
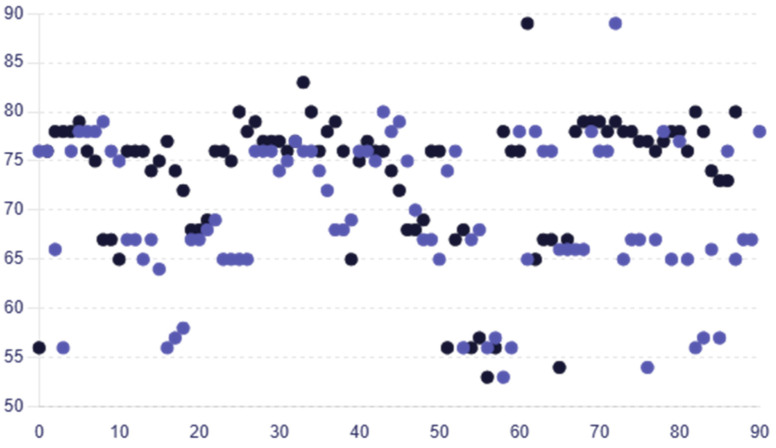
Scatter plot comparing the individual RNFL thickness values for each patient in group A and group B, with colors representing each group (group A—purple circles; group B—black circles).

The average RNFL thickness values for female participants were greater, at 68.31 ± 7.65, than those for male participants, who had an average of 66.33 ± 7.19. The average RNFL thickness values for female participants in group B exhibited the same pattern, being greater than the male average RNFL thickness at 75.28 ± 6.13 versus 72.45 ± 8.21. Age-dependent variations in RNFL thickness were not statistically significant (*p* > 0.05) indicating that there is no important difference in RNFL thickness based on age in the dataset in group A or group B (one-way ANOVA). The RNFL thickness in group A was reduced by 6.09%, with a mean (SD) value of 70.06 ± 6.95 microns compared to 74.60 ± 5.25 microns in group B, indicating a significant reduction in group A. Similar decreasing trends were observed across the different RNFL thickness quadrants. Figure 2 displays individual RNFL thickness values across the two groups.

**Figure 3:**
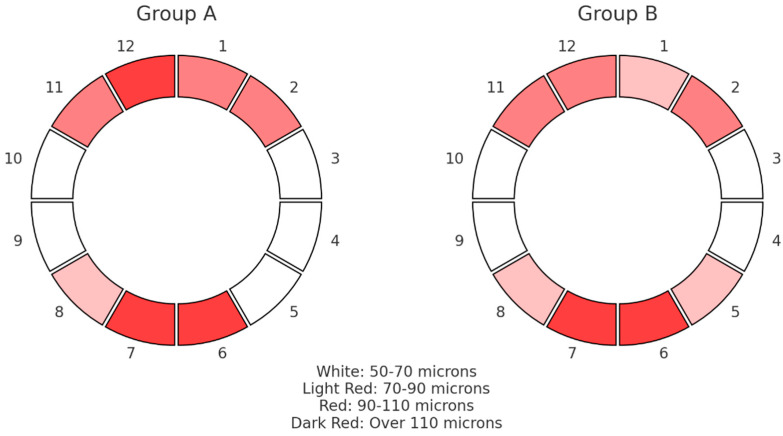
Pie chart that shows the median values of RNFL thickness distribution in microns (μm) for the optic nerve head in 12 clock hours for the two groups.

In Figure 3, the median results of the RNFL thickness pattern for the ONHs are graphically depicted, grouped in clock hours from 1 to 12 for the two groups under examination. The colors represent the RNFL thickness measurements in microns according to their statistical dispersion. There were no appreciable variations in the thickness of the sectoral RNFLs between the groups; both fell within the usual range. Comparing group A with group B, lower average median thickness values were found among inferior segments, while group A exhibited elevated superior RNFL thickness in comparison with group B.

In our analysis, we observed statistically significant differences when comparing ages and RNFL thickness between group A and group B. Specifically, the ages of the patients in the two groups indicated a significant difference between them (*p* < 0.05). Similarly, the RNFL thickness parameter demonstrated a significant difference (*p* < 0.05). Conversely, the IOP did not show a statistically significant difference between the two groups, with *p* > 0.05. The cup-to-disc ratio exhibited a marginally significant difference, with *p* = 0.052, suggesting a potential difference that approaches statistical significance. Table 3 displays IOP and ONH characterization in the two groups.

### 3.3. Clinical Features of Thyroid Eye Disease Patients

**Figure 4:**
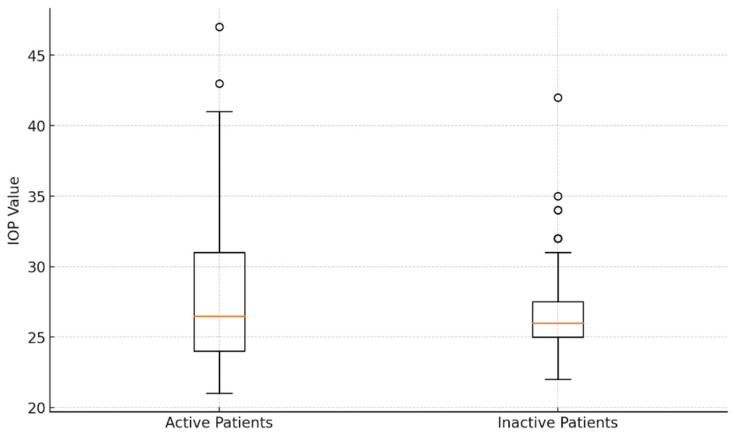
Comparative analysis of intraocular pressure values: box plot visualization for active and inactive patients.

With a CAS ≥ 3, forty-four eyes (48.35%) were active, whereas the other eyes (CAS < 3) were inactive (51.64%). The average IOP in the active eyes was 28.16 ± 5.75 mmHg, which was not statistically different from the inactive eyes’ IOP of 27.02 ± 3.93 mmHg (*p* > 0.05). Figure 4 shows an analysis of introcular pressure of patients with active and inactive disease.

Eyes were classified separately in the EUGOGO classification across all severity levels, comprising mild (28 eyes, 30.76%), moderate–severe (47 eyes, 51.64%), and sight-threatening disease (16 eyes, 17.58%). Furthermore, there was no statistically significant variation in the IOP levels across different system severities, which could mean that there are different mechanisms that could have led to the presence of glaucoma in these patients.

**Figure 5:**
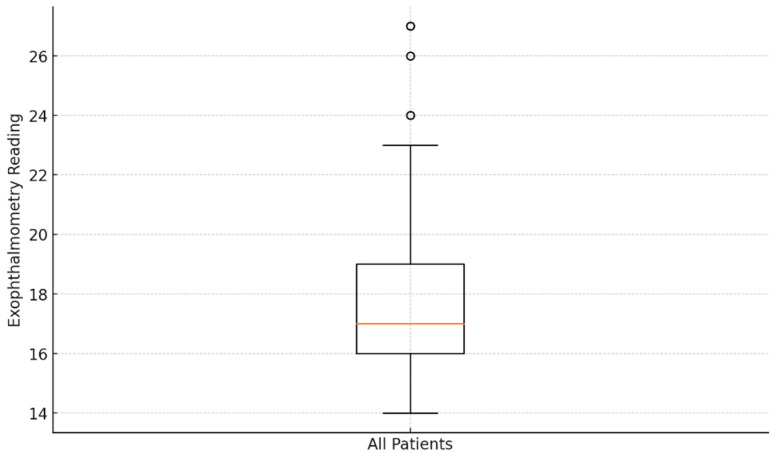
Analysis of exophthalmometry data: box plot visualization.

Figure 5 displays the exophthalmometry data patterns in the eyes of the 53 patients with Basedow–Graves’ disease. The mean exophthalmometry value for all eyes was 18.03 ± 3.15 mm, with a range of 14–27 mm. Analysis revealed no significant difference in the degree of exophthalmos between the left and right eyes (*p* > 0.05). Although IOP tended to increase with greater proptosis, the correlation between IOP and exophthalmometry values was not statistically significant (*p* > 0.05).

## 4. Discussion

Differentiating between glaucomatous optic neuropathy and compressive optic neuropathy in individuals with TED accompanied by glaucomatous symptoms might be challenging. Certain clinical symptoms, like high IOP and ONH parameter impairment, are shared by both TED and OAG. As such, diagnosing glaucoma in patients with TED might be difficult. In the initial stages of diagnosing a patient with glaucoma, when IOP is measured, it is unclear whether the glaucoma preceded the active thyroid disease or if it developed subsequently [10]. Compressive optic neuropathy caused by active TED might present with a variety of optic nerve characteristics, including hyperemia and enlargement of the ONH, diffuse or regional pallor, increased cupping, or even a seemingly normal nerve linked to visual field abnormalities and impaired visual acuity even in the presence of an average IOP [8]. We were able to better characterize OAG patterns in patients with TED by analyzing data on disc morphology, RNFL thinning, and comparing them to individuals with OAG.

Significant correlations between open-angle glaucoma and thyroid disease have been observed in some previous research. However, a number of studies have not discovered a meaningful connection [21]. Therefore, the results of this study are significant because they confirm the hypothesis that thyroid disease could be a separate risk factor for glaucoma based on a cohort from a single center. The patients were selected from a larger database to ensure that key demographics (age, sex) and clinical variables (disease severity) were proportionately represented. Emerging evidence suggests a significant link between thyroid disease and an increased risk of glaucoma [22,23]. Thyroid dysfunction can lead to alterations in IOP, optic nerve damage, and changes in orbital anatomy, all of which are critical factors in the development of glaucoma. The autoimmune nature of Basedow–Graves’ disease can exacerbate inflammatory processes within the eye, contributing to glaucomatous damage [23].

The frequency of OAG in adult populations with thyroid disease has been estimated by several studies conducted in various countries to be between 1.50% and 2.00%; the reason for these fluctuations may be the variation in the time interval between the onset of orbital engagement and the diagnosis of OAG, which can take a few years [5,24,25]. Eyelid retraction, proptosis, periorbital edema, conjunctival redness/chemosis, lid lag, restrictive strabismus, exposure keratopathy, soft tissue involvement, and optic neuropathy are the hallmarks of TED. Several studies on the OAG patient population have revealed distinctive structural alterations in the ONH and in the macular area that are followed by functional loss [25]. Since there is a dearth of information on sectoral optic nerve injury in patients with TED and glaucoma, and the correlation between optic disc damage and elevated IOP has not yet been explored, the second goal of this investigation was to ascertain the relationship between optic disc damage and clinical signs in the TED group.

According to previously released studies, RNFL measurements adhered to the ISNT rule and were thickest in the inferior quadrant, followed by the superior, nasal, and temporal quadrants [26]. This study’s findings are in line with earlier research [27] that found that each ONH quadrant showed consistent thinning of the RNFL and that there was a significant difference in average thickness between each RNFL quadrant and normal eyes. OCT measurements of the peripapillary RNFL thickness yield valid and objective estimates of glaucomatous optic nerve injury [26]. Even in healthy eyes, RNFL thinning can develop with aging at a mean rate of −0.82 microns/year [28]. The advancement of glaucoma is associated with a faster rate of RNFL thinning, ranging from −2.12 μm/year, in comparison to age [27]. Nonglaucomatous optic neuropathies can be distinguished using quadrant and sectoral measurements as well as through the examination of average global RNFL thickness [29]. Singh et al. discovered a statistically significant variation between each RNFL quadrant’s average thickness and normal eyes. In every ONH quadrant in OAG, there is consistent thinning of RNFLs [30]. In line with earlier findings that RNFL loss was most noticeable in the inferotemporal sector in the frequency distribution and progression analysis, other studies also discovered that RNFL defects in glaucoma were most commonly found in the previously mentioned sector, followed by the superotemporal sector [31,32]. Our OCT examination findings regarding RNFL thinning were comparable to those from an earlier study on glaucoma [22]. Using OCT, similar published findings have reported yearly rates of decline at 0.19 microns/year and 0.11 microns/year, respectively [26]. RNFL thinning was most frequently found in the nasal and temporal regions, aligning with other studies’ findings [26,28,30]. Medical attention may be necessary in cases of atypical glaucoma presentations in patients with general RNFL thinning on disc OCT tests and elevated IOP.

In cases of active orbitopathy, elevated IOP is expected, which may have an effect on the development of glaucomatous neuropathy. In the entire population, age, race, high IOP, corneal thinness, high vertical cup-to-disc ratio, and myopia are known risk factors for the development of glaucoma [33]. This study found that the characteristics linked with raised IOP in TED patients were younger age, female patients, and higher disease activity stages (EUGOGO). Thus, it can be said that the TED group had many of the ocular factors that were positive for the development of glaucoma. Also, IOP was relatively consistent across different age ranges and between the two patient groups studied. Higher IOP and cup-to-disc ratio measurements, as well as thinner macula and RNFLs in comparison to the general population, were also identified in some earlier investigations examining alterations in RNFL thickness and ONHs in participants with TED [34]. Recent research has revealed the same risk variables for the onset of elevated IOP: disease activity and length of TED [6,35]. Since RNFL thickness can be the first noticeable structural alteration in glaucoma, it has previously been established that this parameter is essential for early detection [36]. Patients with TED had RNFLs that were generally thinner than the general population’s normal values [37,38]. In individuals with TED who do not have symptomatic optic nerve dysfunction, other investigations have also shown RNFL thinning, especially in the inferior quadrant [10,38]. This raises the prospect that structural RNFL abnormalities might be present regardless of other changes, and that RNFL damage can develop irrespective of IOP. The two groups from our research did not exhibit significantly impaired sectoral RNFL thickness values; however, patients with the greatest IOP levels (≥27 mmHg) all experience sectoral RNFL impairment, according to research. This suggests a favorable association between structural damage to the optic nerve and a noticeably high IOP.

In comparison to group A, the average NRR area in group B was thinner, while the average optic disc volume was greater in group A. These findings suggest that these metrics, as opposed to average RNFL thickness, which only exhibited an unsignificant difference between the groups, may be able to distinguish between TED and glaucoma groups more successfully and identify early alterations in the optic nerve head between them. These findings are consistent with a recent study that found disc cupping to be the most prevalent optic disc characteristic in 51.00% of TED patients with elevated IOP [38].

The percentage of smokers in the first group (56.60%) and in the second group (52.83%) was greater than that of non-smokers, surpassing other countries’ smoking rates [39]. However, it cannot be argued that smoking is linked to higher IOP values and optic neuropathy because there was no statistically significant difference between the two groups. Only the extended duration of active orbital disease resulting in chronically raised IOP was linked to the progression to glaucoma in a study of risk variables for glaucoma in individuals with TED. Age, race, and proptosis were found to be ineffective in predicting glaucoma development in patients, according to Cockerham et al. [11].

There is still much to discover about the role that gender plays in the onset and course of glaucoma. Numerous large-scale epidemiological studies carried out globally have produced inconsistent findings about risk differences between the sexes for developing glaucoma [40,41]. In terms of the gender disparity in TED, women appear to be afflicted 2.5–6 times more frequently than men; in cases of severe illness, however, the ratio of females to males is reversed at 1:4.31; additionally, men had higher elevations in IOP [42]. The difference in illness severity between the sexes may have contributed to our findings, which indicate that female sex increases the risk of glaucoma in TED. The results demonstrate that OAG was associated with older age (≥45 years) and male sex; however, there was no statistical difference between the sexes. It remains unclear whether Basedow–Graves’ disease serves as a direct trigger for the early onset of glaucoma, or if the younger age of onset typical of Basedow–Graves’ disease contributes to the development of glaucoma. This ambiguity highlights the need for further research to elucidate the causal relationship between these conditions.

Effective management of various ocular conditions, such as diabetic retinopathy, age-related macular degeneration, and uveitis, has demonstrated the critical importance of early intervention and tailored treatment strategies, highlighting the need for a comprehensive approach to managing OAG in patients with thyroid eye disease [43,44].

### Limitations

Due to the retrospective nature of our investigation, we were unable to clearly define the relationship between OAG and TED or show the manner in which OAG progresses over time in patients with TED. Although some changes in the orbit linked to Basedow–Graves’ disease may have a role in the development of disease, the findings of our research did not identify the pathophysiologic causes of this disease. The primary issue is the inability to establish temporal relationships, as data are collected at a single point in time. This limitation makes it challenging to determine whether TED contributes to the onset or progression of glaucoma, or if the conditions merely coexist.

Moreover, the study’s cross-sectional nature introduces biases related to sample selection, potentially leading to findings that are not generalizable to the broader population of patients with these conditions. Longitudinal studies would be necessary to overcome these limitations, allowing for a more comprehensive assessment of how TED affects the progression of glaucoma over time, as well as evaluating the severity of the disease and its response to treatment.

## 5. Conclusions

In conclusion, our study highlights the significant impact of TED on the progression and severity of OAG. Patients with both conditions exhibited slightly different disease progression and optic nerve damage compared to those with OAG alone. These findings suggest that TED may exacerbate glaucoma through mechanisms involving elevated IOP and structural changes in the optic nerve. Additionally, demographic factors such as age and gender appeared to influence disease outcomes, underscoring the need for monitoring and management strategies in this patient population. Further research is essential to elucidate the pathophysiological connections between TED and glaucoma, which could inform better prevention and treatment approaches to preserve visual function. 

## Figures and Tables

**Table 1:** Inclusion and exclusion criteria.

	Inclusion Criteria	Exclusion Criteria
Group A (TED + OAG)	Age > 18 IOP ≥ 21 mmHg Signs of ONH impairment Open angle Previously diagnosed Basedow–Graves’ disease Clinical signs of TED (eyelid retraction, proptosis, conjunctival injection, lid lag/lid edema, restricted eye movements)	Age < 18, Refractive error > ±6 diopters and/or astigmatism > ±2 diopters Previous ocular surgery Closed angle on gonioscopy Other autoimmune disorders
Group B (OAG)	Age > 18 IOP ≥ 21 mmHg Signs of ONH impairment Open angle	Age < 18, Refractive error > ±6 diopters and/or astigmatism > ±2 diopters Previous ocular surgery Closed angle on gonioscopy Other autoimmune disorders TED

Abbreviations: IOP—intraocular pressure; OAG—open-angle glaucoma; ONH—optic nerve head; TED—thyroid eye disease.

**Table 2:** General characteristics of patients in group A and B.

	Group A		Group B	
	No.	%	No.	%
Age (years)				
20–30	5	9.43	2	3.77
31–40	17	32.07	5	9.43
41–50	20	37.73	12	22.64
51–60	8	15.09	21	39.62
>60	3	5.66	13	24.52
Gender				
Female	47	88.67	25	47.16
Male	6	11.32	28	52.83
Smoking behavior				
Smoker	30	56.60	28	52.83
Non-smoker	23	43.4	25	27.16
Systemic diseases				
Arterial hypertension	25	47.16	16	30.18
Diabetes mellitus	4	7.54	5	9.43
Other	14	26.41	10	18.86
Thyroid disease classification				
CAS				
Active	44	48.35	-	-
Inactive	47	51.64	-	-
EUGOGO				
Mild	28	30.76		
Moderate–severe	47	51.64		
Sight-threatening disease	16	17.58	-	-

Abbreviations: CAS—clinical activity score; EUGOGO—European Group on Graves’ Orbitopathy.

**Table 3:** OCT findings for the two groups.

Parameter	Group	Min	Max	Centile 25	Centile 75	Mean ± SD
IOP	A	21	47	24	27	27.5 ± 4.90
	B	23	43	24	28	28.1 ± 4.90
RNFL thickness	A	53	80	66	76	70.06 ± 6.95
	B	55	79	68	78	74.60 ± 5.25
Cup-to-disc ratio	A	0.4	0.8	0.5	0.7	0.60 ± 0.15
	B	0.3	0.8	0.5	0.6	0.54 ± 0.18
NRR area	A	0.63	1.12	0.77	1.09	0.92 ± 0.20
	B	0.61	1.18	0.70	1.05	0.91 ± 0.22
Optic disc volume	A	0.151	0.835	0.213	0.574	0.43 ± 0.25
	B	0.112	0.713	0.187	0.490	0.37 ± 0.21

Abbreviations: NRR—neuroretinal rim; RNFL—retinal nerve fiber layer.

## Data Availability

Data is available upon request due to ongoing research.
